# Detection of specific RBD^+^ IgG^+^ memory B cells by flow cytometry in healthcare workers and patients with inborn errors of immunity after BNT162b2 m RNA COVID-19 vaccination

**DOI:** 10.3389/fimmu.2023.1136308

**Published:** 2023-05-04

**Authors:** Lucía del Pino Molina, Luz Yadira Bravo Gallego, Pilar Nozal, Yolanda Soto-Serrano, Ana Martínez-Feito, Keren Reche-Yebra, Andrea González-Torbay, Ricardo Cuesta-Martín de la Cámara, Carla Gianelli, Carmen Cámara, J. González-García, Miguel González-Muñoz, Rebeca Rodríguez-Pena, Eduardo López Granados

**Affiliations:** ^1^ Center for Biomedical Network Research on Rare Diseases (CIBERER U767), ISCIII, Madrid, Spain; ^2^ Lymphocyte Pathophysiology in Immunodeficiencies Group, La Paz Institute for Health Research (IdiPAZ), Madrid, Spain; ^3^ Clinical Immunology Department, La Paz University Hospital, Madrid, Spain; ^4^ Center for Biomedical Network Research on Rare Diseases (CIBERER U754), ISCIII, Madrid, Spain; ^5^ Complement Research Group, La Paz Institute for Health Research (IdiPAZ), Madrid, Spain; ^6^ Immuno-Rheumatology Research Group, La Paz Institute for Health Research (IdiPAZ), Madrid, Spain; ^7^ HIV Unit, Internal Medicine Department, La Paz University Hospital, AIDS and Infectious Diseases Group, Center for Biomedical Network Research on Infectious Diseases (CIBERINFEC CB21/13/00039), La Paz Institute for Health Research (IdiPAZ), Madrid, Spain

**Keywords:** B cells, memory B cells (MBCs), SARS – CoV – 2, vaccine, inborn errors of immunity (IEI), humoral and cellular response

## Abstract

**Introduction:**

Inborn errors of immunity (IEI) are a heterogeneous group of diseases caused by intrinsic defects of the immune system. Estimating the immune competence of immunocompromised patients for an infection risk assessment or after SARS-CoV-2 vaccination constituted a challenge.

**Methods:**

The aim of this study was to determine the humoral responses of patients with IEI through a comprehensive analysis of specific receptor-binding domain-positive (RBD+) IgG+ memory B cells (MBCs) by flow cytometry, together with routine S-specific IgG antibodies and QuantiFERON SARS-CoV-2 (T-cell response), before the vaccine and 3 weeks after a second dose.

**Results and discussion:**

We first analyzed the percentage of specific RBD+ IgG+ MBCs in healthy healthcare workers. Within the control group, there was an increase in the percentage of specific IgG+ RBD+ MBCs 21 days after the second dose, which was consistent with S-specific IgG antibodies.Thirty-one patients with IEI were included for the pre- and post-vaccination study; IgG+ RBD+ MBCs were not evaluated in 6 patients due to an absence of B cells in peripheral blood. We detected various patterns among the patients with IEI with circulating B cells (25, 81%): an adequate humoral response was observed in 12/25, consider by the detection of positive S-specific IgG antibodies and the presence of specific IgG+ RBD+ MBCs, presenting a positive T-cell response; in 4/25, very low S-specific IgG antibody counts correlated with undetectable events in the IgG+ RBD+ MBC compartment but with positive cellular response. Despite the presence of S-specific IgG antibodies, we were unable to detect a relevant percentage of IgG+ RBD+ MBCs in 5/25; however, all presented positive T-cell response. Lastly, we observed a profound failure of B and T-cell response in 3 (10%) patients with IEI, with no assessment of S-specific IgG antibodies, IgG+ RBD+ MBCs, and negative cellular response. The identification of specific IgG+ RBD+ MBCs by flow cytometry provides information on different humoral immune response outcomes in patients with IEI and aids the assessment of immune competence status after SARS-CoV-2 mRNA vaccine (BNT162b2), together with S-specific IgG antibodies and T-cell responses.

## 1 Introduction

Severe acute respiratory syndrome coronavirus 2 (SARS-CoV-2), a new coronavirus discovered in 2019, resulted in the COVID-19 pandemic (1). Within a remarkably short time, however, research and clinical laboratories worldwide were able to gain knowledge on the natural immunological response to SARS-CoV-2 (2–4), in which several innate and adaptative mechanisms are implicated, and subsequently on the response to the newly developed vaccines. Estimating the immune competence of immunocompromised patients for an infection risk assessment constituted a challenge for clinical immunology units (5), which was particularly relevant for patients with inborn errors of immunity (IEI) (6), a large group of entities affecting a myriad of effector immune responses, with B-cell defects being the most prevalent (7).

Several immune mechanisms are involved at various levels in defending against SARS-CoV-2 (8). Antibody production by B cells is a critical component for adaptive immune responses (9). Naïve B cells recognize antigens from native proteins of infectious agents or proteins encoded in vaccine vectors, diversifying the response in secondary lymphoid organs. In the germinal centre (GC) reaction memory B cells (MBCs) are generated, with high affinity and specificity (due to the process of somatic hypermutation) and with different immunoglobulin heavy chain isotypes (through class-switch recombination) (10). MBCs differentiate into plasmablasts producing neutralizing antibodies. Thanks to the generation of MBC through subsequent exposure to the antigen by infection or vaccine boosters, the antibody responses are much faster and more efficient (11).

BNT162b1 is a lipid-nanoparticle-formulated, nucleoside-modified mRNA vaccine that encodes the trimerized receptor-binding domain (RBD) of the SARS-CoV-2 spike glycoprotein. In Spain, the first group to receive the SARS-CoV-2 mRNA vaccine (BNT162b2/Pfizer) was healthcare workers. Weeks later, immunocompromised patients began to receive the same vaccine.

IEI are a heterogeneous group of diseases caused by intrinsic defects of the immune system (5). Here, we report patients from various IEI categories (7), including combined immunodeficiency (CID) affecting humoral and cellular immunity: CD40 ligand deficiency (CD40L), CID associated with syndromic features: Wiskott-Aldrich syndrome (WAS) with congenital thrombocytopenia and DiGeorge syndrome (thymic defect with additional congenital anomalies). Predominant antibody deficiencies: Bruton tyrosine kinase (BTK deficiency or X-linked agammaglobulinemia), Common variable immunodeficiency with no gene defect specified (CVID), activated p110 d syndrome (APDS), CD19 deficiency, NFKB1 deficiency, NFKB2 deficiency, and isolated immunoglobulin (Ig) G subclass deficiency with IgA deficiency. Diseases of immune dysregulation: CTLA-4 haploinsufficiency (regulatory T-cell defects) and autoimmune polyendocrinopathy-candidiasis-ectodermal dystrophy (APECED). And congenital defects of phagocyte: X-linked granulomatous disease (CGD); as well as 2 patients with suspected IEI and 1 patient with a secondary antibody deficiency after rituximab therapy.

The aim of this study was to determine the humoral responses of the patients with IEI through a comprehensive analysis of specific RBD^+^ IgG^+^ memory B cells by flow cytometry, together with routine S-specific IgG antibodies and QuantiFERON SARS-CoV-2, before the vaccine and 3 weeks after the second dose, compared with healthy donors (HD).

## 2 Materials and methods

### 2.1 Samples

Initially, 10 donor samples from healthcare workers were obtained to validate the technique, 5 with positive antibody assessment for SARS-CoV-2 and another 5 donors with no suspected previous SARS-CoV-2 infection.

Within the IEI cohort at La Paz University Hospital, 31 patients from various IEI groups were included to analyze specific RBD^+^ IgG^+^ memory B cells by flow cytometry after the administration of the SARS-CoV-2 mRNA vaccine (BNT162b2/Pfizer).

After each participant had granted their informed consent, blood samples were obtained according to the principles of the Declaration of Helsinki. The study was approved by the local ethics committee at Hospital Universitario La Paz (PI-4101).

The clinical data were obtained from the medical records updated during routine medical visits for the diagnosis and follow-up at the outpatient clinic of the Clinical Immunology Department.

### 2.2 Flow cytometry identification of B cells and their subsets in blood

Specific B cells and their subsets were identified by flow cytometry in EDTA anticoagulant blood samples from patients and HD. We used the EuroFlow BulkLysis and stain technique (12, 13). Briefly, 2 mL of blood was diluted in *Bulklysis* solution (Cytognos SL, Salamanca, Spain), incubated for 15 min at room temperature. The nucleated cells were centrifuged for 10 min at 800 g, and washed twice with PBS containing 0.5% BSA and 0.09% NaN_3_ (800 g and 540 g respectively, for 5 min). The cells were subsequently resuspended at 5×10^5^ nucleated cells/µL, and a total of 150 µL of cells (7.5 x10^6^ cells) were stained with the corresponding surface antibodies (**Table S1**) for 30 min in the dark. The RBD protein was manufactured and conjugated to PE fluorochrome by Immunostep. Then, the cells were fixed with 2 mL of FACS lysing solution (BD Biosciences) for 10 min and finally washed with PBS+0.5%BSA+0.09 NaN_3_. Instrument setup and calibration were performed prior to data acquisition on ≥1×10^6^ cells (range, 1×10^6^ – 5×10^6^ cells) in DxFLEX flow cytometers (Beckman Coulter). Data analysis was performed on pseudo-anonymized flow cytometry standard (FCS) data files using FlowJo and Infinicyt software (Cytognos SL, Salamanca, Spain).

For the data analysis, a standardized gating strategy was employed to identify all B-cell subsets (**Supplementary Figure 1A**). Total B cells were gated first after exclusion of debris and cell doublets (FSC-A/FSC-H), then by their low-to-intermediate forward (FSC) and sideward (SSC) light scatter properties and for the positive staining for CD19. B cells (CD19+) were subclassified into 5 different subsets based on their staining profile for CD38, CD27, smIgM, smIgA, smIgG, and smIgD: a) CD27^-^ CD38^-^ smIgM^+^IgD^+^ mature naive B cells; b) CD27^+^ CD38^lo^ smIgM^++^D^+^ (MD^+^) unswitched MBCs; c) CD27^+^ CD38^lo^ smIgM^-^D^-^IgG^+^ switched MBCs; d) CD27^+^ CD38^lo^ smIgM^-^D^-^IgA^+^ switched MBCs; and e) CD27^++^ CD38^hi^ plasmablasts/PCs smIgM^-^D^-^ and IgM^+^ (**Supplementary Figure 1A**). We then analyzed the percentage of RBD^+^ CD38^lo^ smIgM^-^D^-^IgG^+^ switched MBCs (hereafter, specific RBD^+^ IgG^+^ MBCs), considering that only when more than 10 events were recorded in the positive gate, the percentages of specific RBD^+^ IgG^+^ MBCs are expressed within the total IgG^+^ MBCs. For the patients with a CD19 defect, a CD20 antibody was used to analyze total B cells and subsets.

### 2.3 SARS-CoV-2 IgG antibodies and QuantiFERON SARS-CoV-2 assay

We analyzed, as part of the routine diagnostic two assays approved with vitro diagnostic regulation (IVDR), the determination of SARS-CoV-2 IgG antibodies and QuantiFERON SARS-CoV-2 assay in parallel to the identification of specific RBD^+^ IgG^+^ MBCs by flow cytometry.

SARS-CoV-2 IgG antibodies were detected in serum samples from the HD and the patients with IEI with EliA SARS-CoV-2-Sp1 IgG (Thermo Fisher Scientific) according to the manufacturer’s instructions. The technique’s measurement range is 0.7–204 U/mL. After the establishment of the first IgG anti-SARS-CoV-2 standard by World Health Organization National Institute for Biological Standards and Control (WHO 20/136), a correlation study was performed in which 1 EliA unit corresponded to 4 binding antibody units (BAU)/mL from the World Health Organization standard.

The QuantiFERON SARS-CoV-2 (QIAGEN) assay was performed for the patients with IEI, following the manufacturer’s recommendations. Of note, this technique was not commercially available during the vaccination study of healthcare workers at the beginning of 2021. QuantiFERON SARS-CoV-2 assay is an *in vitro* diagnostic test designed for the qualitative detection of interferon-γ (IFN-γ) produced by CD4+ and CD8+ T cells in response to stimulation by a SARS-CoV-2 peptide cocktail in whole blood. Whole blood is collected in specific commercial tubes (Qiagen) which contains a combination of specific peptide SARS-CoV-2 antigens (Ag 1 and Ag2) to stimulate lymphocytes involved in cell mediated immunity. Also another’s tubes called Nil tube and mitogen tube are used as negative and positive controls. The commercial tubes are incubated 16-24 h at 37°C, then tubes are centrifuged and plasma from stimulated samples are harvested. After that an automatic enzyme-linked-immunoabsorbent assay (ELISA) is performed to measure IFN γ. Positive responses were assessed with a cut-off of >0.15 IU/mL, according to the manufacturer’s instructions and as reported elsewhere (8).

### 2.4 Statistics

The data was analyzed using GraphPad Prism version 9.0 software (San Diego, CA, USA). Statistical differences between the patients with IEI and the healthy donors were determined with the Mann–Whitney U test (for continuous variables) or Fisher’s exact and chi-squared tests (for categorical variables). P-values <0.05 were considered to have statistical significance and were coded as follows: *p <0.05; ** p <0.01; ***p <0.001; and, **** p <0.0001.

## 3 Results

### 3.1 Validation of identification of specific RBD^+^ IgG^+^ MBCs by flow cytometry

**Figure 1:**
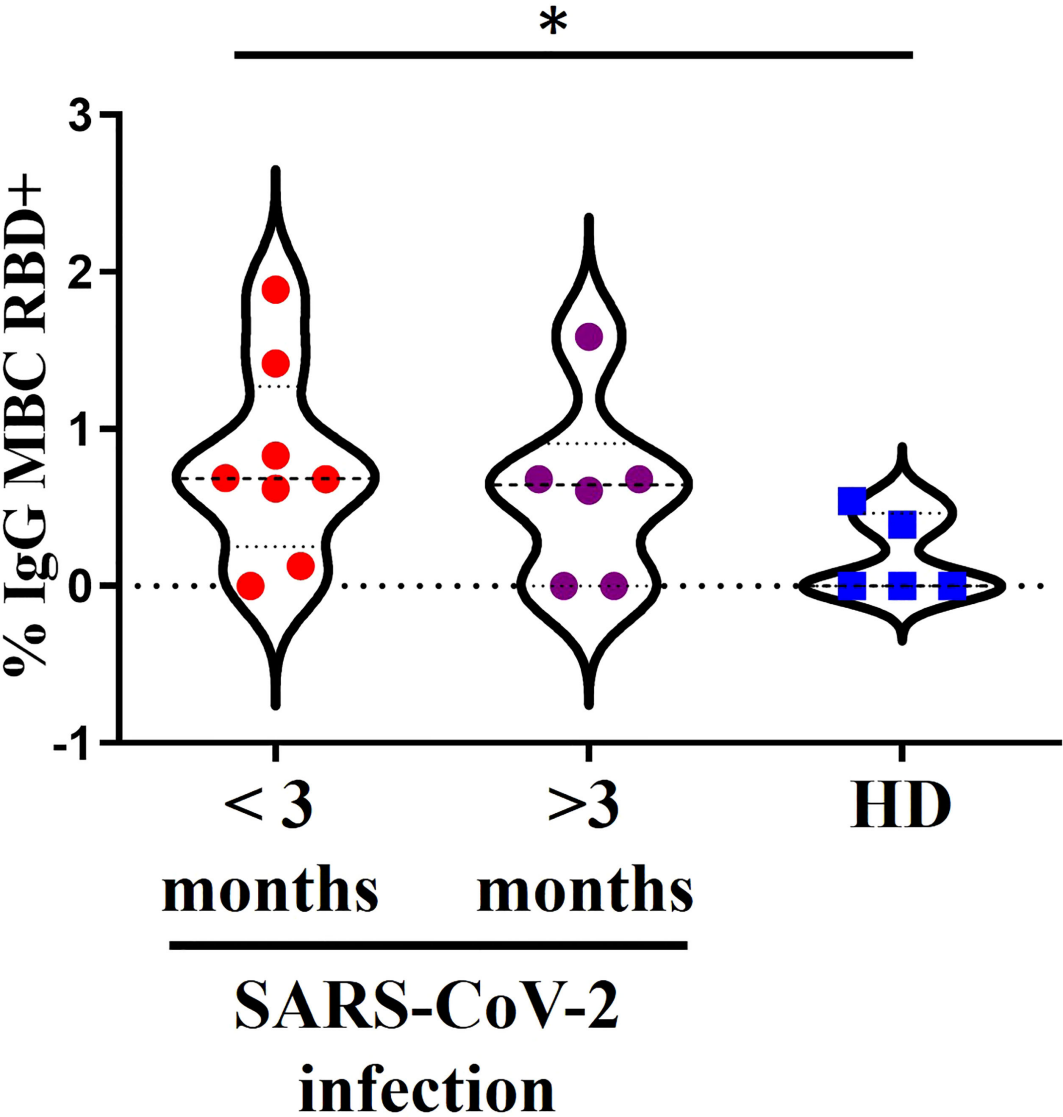
A 15 donors with a positive assessment of SARS-CoV-2 infection divided into 2 groups. In red, past infection (>3 months) or recent infection (<3 months) in purple and in blue another 5 donors without documentation of pre-existing SARS-CoV-2 immunity or suspicion of previous infection. *<0.05.

We validated the assay for identification of specific RBD^+^ IgG^+^ MBCs by flow cytometry upon vaccination by estimating the percentage of conjugated RBD^+^ cells in various B-cell subsets from 15 donors (**Figure 1**). This group included 2 subsets of individuals: one with antecedents of SARS-CoV2 infection, either past (>3 months) or recent infection (<3 months); and the other with no evidence of preexisting SARS-CoV-2 immunity or previous infection.

We detected specific RBD^+^ IgG^+^ MBCs in the individuals with a positive assessment of SARS-CoV-2 infection (**Figure 1**). There were no differences between the groups with recent or past infection. Moreover, there was a higher percentage of specific RBD^+^ IgG^+^ MBCs in the individuals with a history of recent SARS-CoV-2 infection (<3 months) compared with the HD no previously infected (p=0.0311) and higher levels in the individuals with past infection, although the difference was not significant in this case.

We detected no specific RBD^+^ IgG^+^ MBCs in 1 individual with a recent history of SARS-CoV-2 infection (<3 months) and in 2 individuals with past infection by SARS-CoV-2. In 2/5 HD with no assessment of natural SARS-CoV-2 infection, a small percentage of specific RBD^+^ IgG^+^ MBCs were detected, but after the second dose this percentage exceeds the titers found prior to vaccination (**Supplementary Figure 2**).

### 3.2 Demographics of the patients with inborn errors of immunity

We selected 31 representative patients with various IEI conditions (19 men and 12 women, median age 33 years, range 16–61 years), as well as 25 HD (healthcare workers) unrelated to the patients (5 men and 20 women, median age 48 years, range 22–64 years).

Among the 31 patients with IEI, 3 reported previous SARS-CoV-2 infection (positive PCR), and another 4 had positive S-specific IgG antibodies prior to vaccination.

**Supplementary Table 2** details the baseline and clinical characteristics of the IEI cohort included in the study, with IEI diagnosed according to IUIS classification (7).

### 3.3 Humoral immune response in patients with inborn errors of immunity

To determine the humoral response after SARS-CoV-2 mRNA vaccination (BNT162b2/Pfizer) in 31 patients with IEI, we analyzed the specific IgG^+^ RBD^+^ MBCs together with S-specific IgG antibodies compared with 25 HD prior to vaccination and 21 days after the second dose.

**Figure 2:**
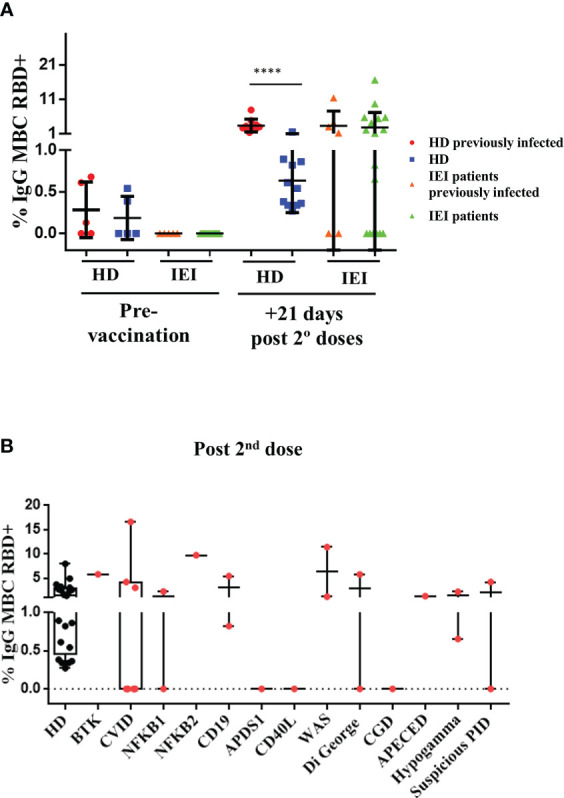
Analysis of specific RBD^+^ IgG^+^ MBCs. **(A)**: Percentage of specific RBD^+^ IgG^+^ MBCs (within total IgG^+^ MBC) prior to vaccination and 21 days after the second dose in healthy healthcare workers (HD) and patients with inborn errors of immunity (IEI). In red, HD with previous natural SARS-CoV-2 infection; in blue, non-infected HD; in orange, patients with previous natural SARS-CoV-2 infection; and in green, patients non-previously infected, prior to vaccination, and 21 days after the second dose. **(B)**: Percentage of specific RBD^+^ IgG^+^ MBCs (within total IgG^+^ MBCs) 21 days after the second dose, in healthcare workers (HD) and in patients with IEI classified in different IEI categories. ****<0.0001.

Within the HD group, those with a positive assessment of previous SARS-CoV-2 infection and those with no assessment of natural SARS-CoV-2 infection presented an increase in the percentage of specific IgG^+^ RBD^+^ MBCs 21 days after the second dose, although the percentage of specific IgG^+^ RBD^+^ MBCs was higher in the group with previous SARS-CoV-2 infection (p<0.0001) (**Figure 2A**).

In the IEI group, 31 patients were included for the pre- and post-vaccination study. IgG^+^ RBD^+^ MBCs could not be evaluated in 6 of these patients due to an absence of B cells in peripheral blood. The percentage of specific IgG^+^ RBD^+^ MBCs was considered when at least 10 events were recorded. We detected no reliable percentage of specific IgG^+^ RBD^+^ MBCs in any of the patients with IEI in the pre-vaccination study. In the post-vaccination study performed 21 days after the second dose, however, we detected heterogeneity among the patients with IEI, as expected. Some patients presented similar percentages of specific IgG^+^ RBD^+^ MBCs compared with the HD, while in others we were unable to detect any specific cells (**Figures 2A, B**).

There were differences between the patients with IEI who presented a previous natural SARS-CoV-2 infection and underwent the complete vaccination schedule and the patients with IEI with no previous natural infection. Of the 7 patients with IEI with a positive assessment of previous SARS-CoV-2 infection (and circulating B cells), 3 reached a similar percentage of specific IgG^+^ RBD^+^ MBCs as the HD with previous natural infection and a complete vaccination schedule. Another 4 patients failed to produce (or at least we were unable to detect) any specific IgG^+^ RBD^+^ MBCs, and in other 1 patient the specific IgG^+^ RBD^+^ MBCs were not evaluated due to the absence of B cells in peripheral blood.

**Figure 3:**
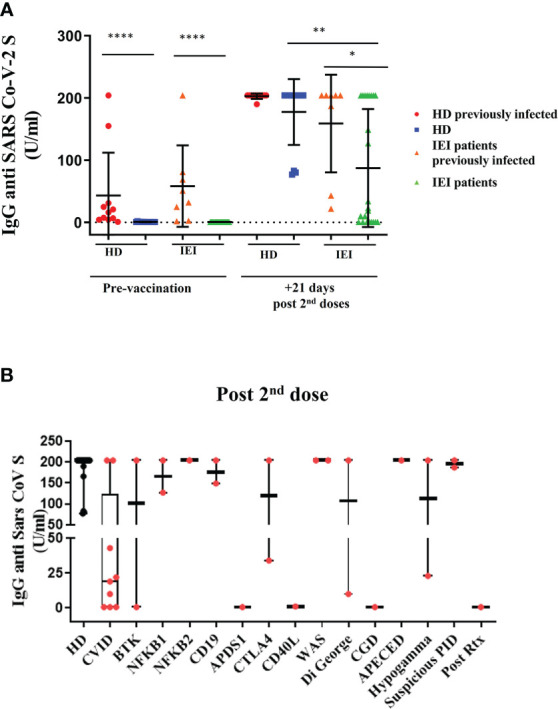
Plasma antibody dynamics against SARS-CoV-2. **(A)**: Results of ELISAs measuring plasma reactivity Spike protein (S-specific IgG antibodies) pre-vaccination and 21 days post second dose in healthy healthcare workers (HD) and patients with inborn errors of immunity (IEI). In red, HD with previous natural SARS-CoV-2 infection; in blue, non-infected HD; in orange, patients with previous natural SARS-CoV-2 infection; and in green, patients non-previously infected. **(B)**: S-specific IgG antibodies 21 days after the second dose, in healthcare workers (HD) and in patients with IEI classified in different IEI categories. *p <0.05; **p <0.01; ****p <0.0001.

This result is consistent with S-specific IgG antibodies quantification; the HD and patients with IEI were clearly differentiated between those with previous natural infection and those with none (p<0.0001) (**Figure 3A**). In fact, the pre-vaccination determination of antibodies in non-previously infected HD and patients was below the technique’s detection limit (>0.7 U/mL). The concentrations of SARS-CoV-2 antibodies from the previously infected participants ranged from 0.9 to 204 U/mL. After vaccination, all HD antibody titers increased above 77 U/mL and did not significantly differ between previously infected and non-infected donors. However, there was heterogeneity once again in the IEI group in the production of S-specific IgG antibodies because of the intrinsic nature of their own immune defects (**Figures 3A, B**).

In fact, 21 days after the second dose, the patients with IEI with an assessment of previous SARS-CoV-2 infection presented impaired production of S-specific IgG antibodies compared with the HD with previous natural infection (p-value=0.045). This difference was even more noticeable between the patients with IEI and the HD without an assessment of previous SARS-CoV-2 infection (p<0.0001) (**Figure 3A**). The healthy participants with no previous history of natural infection reached similar S-specific IgG antibody levels as those who were previously infected. Within the IEI group, the patients with a previous history of natural infection presented slightly higher S-specific IgG antibody levels than those patients naïve for SARS-CoV-2 infection (p=0.0482).

**Table 1** shows a summary of the results: green represents a positive result; red corresponds to a negative result; and yellow represents those patients with low titers of S-specific IgG antibodies (range 2–43 U/mL).

Table 1Summary table of results.

**Table:** 

ID	£11	S specific IgG Antibodies		Specific RBD+ IgG+ MBC		QuantiFERON®	
		pre	post	pre	post	pre	post
1	Btk					2	
2	Btk					3	
16	CVID	2	22				
19	CVID						
20	CVID						
22	CVID						
24	CVID						
25	CVID		10				
28	CVID		19				
30	CVID	8.1	43				
36	CVID	5. 1					
7	Nf KBl						
31	Nf KBl	2.7					
10	Nf KB2						
21	CD19						
23	CD19						
6	APDSl						
8	CTLA4		34				
11	CTLA4 +TPH						
9	CD40L						
12	WAS						
27	WAS	***3.2***					
26	Di George		10				
29	Di George						
4	CGD						
5	APECE D	6.9					
13	IgG and IgA hypogamma		***23***				
35	IgG and IgA hypogamma						
32	PID suspicious	***25***					
34	PID suspicious						
33	Post Rtx

**Table:** 

Antibodies		IgG Memory B Cells RBD+		QuantiFERON	
	Negative <O .7		Negative		!Negative
	Positive		Positive		Positive
	Mild positive		No B cells		N.E
			N. E		

Patients with IEI classified into different categories according to their immunologic defect. The results of the 3 techniques: S-specific IgG antibodies, specific RBD^+^ IgG^+^ MBCs analyzed by flow cytometry, and QuantiFERON SARS-CoV-2. Positive results are represented in green (>50 U/mL for S-specific IgG antibodies, relevant percentage of specific RBD^+^ IgG^+^ MBCs when more than 10 events were recorded, and QuantiFERON^®^ SARS-CoV-2 positive response for >0.15 IU/mL); in red, negative results; and in yellow, for those patients with low titers of S-specific IgG antibodies ranging from 2 to 43 U/mL. (N.E., not evaluated).

In all the samples from HD the humoral response after the second dose of the BNT162b2/Pfizer vaccine, the presence or increment of S-specific IgG antibodies is accompanied by the detection of specific IgG^+^ RBD^+^ MBCs. However, heterogeneity was found within the IEI group (**Figure 3B**). In the patient subgroup with no detectable B cells in peripheral blood (6/31, 19%), 2 patients surprisingly had detectable circulating antibodies after natural SARS-CoV-2 infection and presented a adequate T-cell response assessed by positive QuantiFERON SARS-CoV-2 test (**Table 1**), whereas the other 4 patients with IEI failed to produce antibodies due to the absence of B cells, as expected. However, all 4 patients presented a positive QuantiFERON SARS-CoV-2 test.

**Figure 4:**
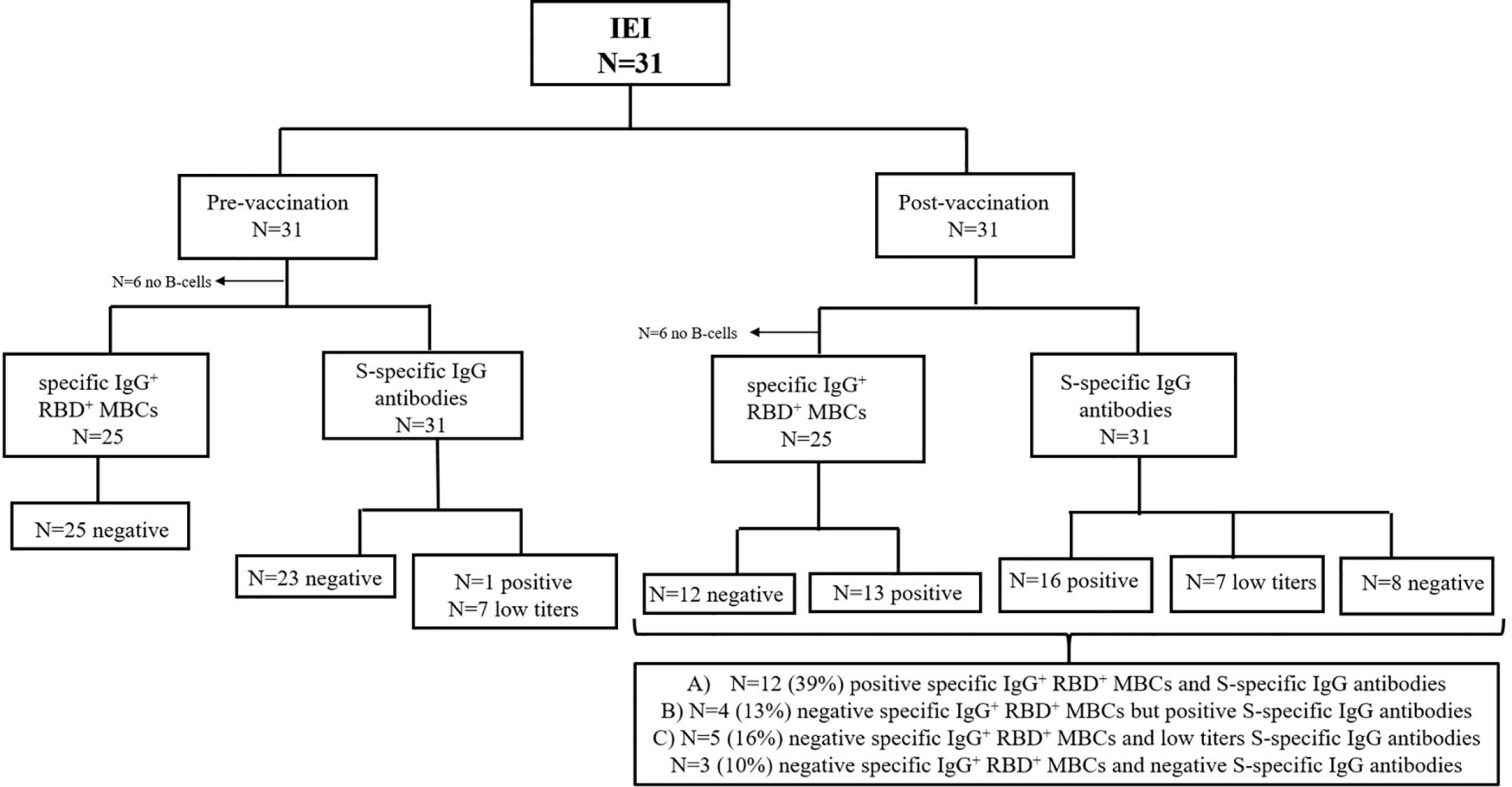
Flow diagram for the analysis humoral response of patients with IEI (n=31). Analysis of specific RBD^+^ IgG^+^ MBCs and S-specific IgG antibodies prior to vaccination and 21 days after the second dose. *<0.05.

In subgroup of IEI patients with circulating B cells (25, 81%), we detected different patterns (**Figure 4**): A) In 12/25 (39%), a good humoral and cellular response could be detected with the presence of S-specific IgG antibodies, together with specific IgG^+^ RBD^+^ MBCs, presenting a positive QuantiFERON SARS-CoV-2 (except for 1 patient); B) 4/25 (13%) had very low S-specific IgG antibody levels (in yellow in **Table 1**), we did not detect enough events in the IgG^+^ RBD^+^ MBCs compartment, but presented a positive QuantiFERON SARS-CoV-2 (except for 1 patient); C) Despite the presence of S-specific IgG antibodies, we were unable to detect a relevant percentage of IgG^+^ RBD^+^ MBCs in 5/25 (16%); but all presented a cellular response with positive QuantiFERON SARS-CoV-2. In another patient with CVID who had malignancy complications, we detected a relevant percentage of specific IgG^+^ RBD^+^ MBCs, it seems there was insufficient time to differentiate into antibody-secreting plasma cells, and the S-specific IgG antibody levels were negative and with impaired T-cell response. Lastly, there was profound failure of B and T-cell response in 3 (10%) of the patients with IEI, with no assessment of S-specific IgG antibodies, IgG^+^ RBD^+^ MBCs, and negative QuantiFERON SARS-CoV-2 results.

**Figure 5:**
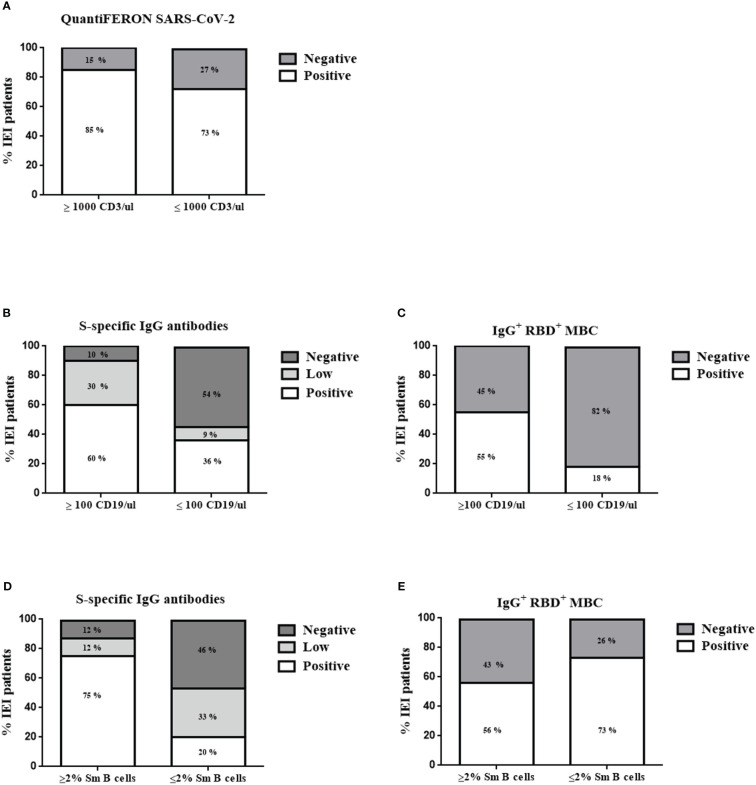
Patient stratification results 21 days after the second dose for QuantiFERON SARS-CoV-2 **(A)** according to CD3 T cells/µL counts, S-specific IgG antibodies **(B, D)**, and specific RBD^+^ IgG^+^ MBCs **(C, E)** according to CD19 B cells/µL counts and percentage of switched memory B cells (Sm B cells), respectively. *<0.01.

We then analyzed whether the absolute counts of total T cells or B cells were associated with a reliable cellular or humoral response. In the patients with IEI with <1000 CD3 T cells/µL, only 27% presented a negative result for the QuantiFERON SARS-CoV-2 (73% of these patients presented a positive cellular response). Among the patients with IEI with >1000 T cells/µL, 85% responded properly to the cellular test, with a positive QuantiFERON SARS-CoV-2 result after the second dose (**Figure 5A**). There were no statistically significant differences. Thus, absolute CD3 T-cell counts appear to not be related to QuantiFERON SARS-CoV-2 results.

When comparing the absolute counts of total CD19 B cells and the humoral response in the patients with IEI with <100 CD19 B cells/µL, we detected no specific IgG^+^ RBD^+^ MBCs in 82% of them (although there were no statistically significant differences; p=0.06). In terms of reduced total B-cell counts (<100 CD19 B cells/µL), 36% of the patients produced detectable S-specific IgG antibodies, and 9% presented a positive result with low titers (**Figures 5B, C**). Ninety percent of the patients with IEI with more than 100 CD19 B cells/µL had the ability to produce S-specific IgG antibodies (30% with low titers). However, having more than 100 CD19 B cells/µL was insufficient for a proper detection of specific IgG^+^ RBD^+^ MBCs in all patients, given their antibody deficiency or immunodeficiency. We were able to detect these cells in only 55% of those patients. Thus, the presence of B cells in peripheral blood of at least >100 CD19 B cells/µL is critical for a good humoral response in terms of S-specific IgG antibodies. There were statistically significant differences between having more or fewer than 100 CD19 B cells/µL and the presence of S-specific IgG antibodies (p=0.0224).

Another typical value used for classifying patients with antibody deficiencies is the percentage of switched memory B cells (Sm B cells) (14). Eighty-seven percent of the patients with IEI with more than 2% of Sm B cells presented S-specific IgG antibodies (12% of them with low titers). Forty percent of the patients with IEI with reduced Sm B cells (<2%) could not produce S-specific IgG antibodies, and we were unable to detect enough specific IgG^+^ RBD^+^ MBCs in 73% of the patients (**Figures 5D, E**). These results indicate that the percentage of Sm B cells influences the presence of detectable S-specific IgG antibodies (p=0.0265).

## 4 Discussion

Since 2020, laboratories worldwide have stepped up their efforts in the fight against the COVID-19 pandemic. Our laboratory, specialized in the B-cell dysregulation in IEI, enabled us to apply our knowledge of B-cell biology through multiparametric flow cytometry (15) to analyze the specific B cells generated by natural SARS-CoV-2 infection and after immunization with the SARS-CoV-2 mRNA vaccine (BNT162b2/Pfizer) in HD and patients with IEI, together with routine well-standardized techniques such as EliA SARS-CoV-2-Sp1 IgG and QuantiFERON SARS-CoV-2.

Our first attempt to establish a multiparametric flow cytometry panel to dissect the specific RBD^+^ IgG^+^ MBCs was run with healthcare workers and patients recruited in the internal medicine department for evaluation of past SARS-CoV-2 infection, together with unexposed healthcare workers. We found a higher percentage of specific RBD^+^ IgG^+^ MBCs in the participants with a history of recent SARS-CoV-2 infection compared with the non-infected HD, as previously reported (16).

In 2/5 HDs without clinical evidences of natural SARS-CoV-2 infection, a small percentage of specific RBD^+^ IgG^+^ MBCs was detected. We cannot formally conclude the presence of some unspecific background binding in our assay, or if this could be reflecting some common coronavirus cross-reactive memory B cells generated in response to previous human common coronaviruses. T-cell specific responses have been described as directed mainly to the S2 domain (17), as reported elsewhere (18–20) although Rodda et al. (16) also detected in HD RBD+ IgM+IgD+ MBCs that could be cross-reactive MBCs generated in response to a previous human coronavirus. Moreover, the cross-reactivity to SARS-CoV-2 T-cell epitopes in unexposed humans could explain the high heterogeneity in infected individuals (21).

In the last days of 2020, a vaccination scheme was started in Spain, and the first group to be vaccinated were healthcare workers. We designed the recruitment of non-previously exposed healthcare workers and others with a history of past SARS-CoV-2 infection to analyze specific B cells by flow cytometry, generated after immunization with the SARS-CoV-2 mRNA vaccine (BNT162b2/Pfizer), together with S-specific IgG antibodies shown by ELISA. Our results indicated that the antibody levels in those individuals with a past infection were higher than those of infection-naïve individuals after the first dose, in line with several previous studies that reported the strongest humoral (20) and cellular T-cell responses (22). Although QuantiFERON SARS-CoV-2 was not commercially available during the vaccination study of healthcare workers, cellular response was measured by the secretion of interleukin-2 and interferon-gamma by the specific CD4^+^CD154^+^Th1 cells (22).

S-specific IgG antibody levels were similar after the second dose in those previously exposed and those unexposed to SARS-CoV-2 infection, as reported in another cohort of healthcare workers (23). After the second dose of the vaccine, we also detected a significantly higher frequency of specific IgG^+^ RBD^+^ MBCs in those participants with previous natural SARS-CoV-2 infection. However, both groups presented an increase in the percentage of specific IgG^+^ RBD^+^ MBCs 21 days after the second dose.

Spike-specific memory B-cell clones can be detected for up to 6 and 8 months in individuals with mild to severe SARS-CoV-2 infection (22), most of which are IgG, whereas IgA is severely reduced, and IgM clones tend to disappear earlier (1). In naturally infected individuals, polyfunctional SARS-CoV-2 antigen-specific B and T memory cells have been detected up to 6 months after the infection (24). B-cell biology also appears to be after the different outcomes in the infectious process, the greater frequency of IgM^+^ memory B cells specific to SARS-CoV-2 was related to less extend symptoms (25).

Humoral immune response is in continuous evolution, with memory B cells starting clonal turnover at approximately 6 months (9). From that moment on, the evolution of the response occurs in the GC where antigens are retained by dendritic cells, and RBD memory B cells undergo greater somatic hypermutation (SHM), being able to produce a broader neutralizing response capacity (9). Prior knowledge from the influenza vaccine indicates that the specific antibody levels are promoted by plasma cells in bone marrow after 4 weeks and decrease after 1 year (26). Another study analyzed the lymph nodes after influenza vaccination and found that the vaccine-binding B-cell clones during the following weeks after vaccination were similar to those in peripheral blood and secondary lymphoid organs, with a high frequency of SHM and broad cross-reactivity. However, the GC B-cell clones in lymph nodes are highly specialized and can target new epitopes and broaden the spectrum of vaccine-induced protective antibodies (11).

Our previous study of healthcare workers provided a reference to compare the patients with IEI, who were included in the vaccination schedule months later. Patients with IEI are characterized by impairment of one or more components of the immune system, depending on their intrinsic defect (7). In general, the risk factors affecting mortality and comorbidity in these patients are similar to those of the general population, and approximately 30% of patients with IEI presented with mild COVID-19 disease (5). In fact, the 3 confirmed patients included in our study had mild COVID-19, and the other 4 patients who had low titers of specific antibodies in the pre-vaccination study might have been asymptomatic. Other cohorts have found a greater comorbidity in patients with primary immunodeficiency and especially in patients with symptomatic secondary immunodeficiency (SID) (27).

We detected a proper humoral and cellular response in 39% of the patients with IEI when analyzing with presence of S-specific IgG antibodies together with specific IgG^+^ RBD^+^ MBCs and a positive QuantiFERON SARS-CoV-2 (with the exception of 1 patient with IgG and IgA hypogammaglobulinemia). Thirteen percent of patients with IEI studied, who had very low S-specific IgG antibodies, had insufficient events in the IgG^+^ RBD^+^ MBC category (**Figure 4**); however, they had a positive QuantiFERON SARS-CoV-2 (except for 1 patient). T-cell response was positive in all but one of the study patients with CVID after vaccination, with similar results in other cohorts (28) and in studies reporting natural SARS-CoV-2 infection (29).

If we consider only the positive humoral response by measuring antibodies, 74% of the patients with IEI were able to generate antibodies (in low and high titers), results similar to those obtained by Delmonte et al., with 85% of patients with IEI producing antibodies after the second dose (30). A pilot study with 11 patients with IEI detected seroconversion after vaccination, except for 1 patient with concomitant immunosuppression therapy (31). Another cohort with 41 patients with IEI showed variability in humoral response in CVID, which was totally impaired in XLA patients but with almost normal T-cell response (32). Reports have highlighted the importance of studying patients with antibody deficiency in an isolated manner due to each patient’s variability and unique characteristics in terms of cellular and humoral immunity (32). In fact, our cohort had a patient with a leaky BTK mutation (in press) that generated a powerful B and T-cell response.

Within the IEI classified as predominant antibody deficiency, we detected specific IgG^+^ RBD^+^ MBCs in half of them, with the exception of 1 patient with CVID, 1 patient with NFKB1 deficiency, 1 patient with NFKB2 deficiency, and 1 patient with suspected IEI without confirmed genetic diagnosis. However, those patients without detectable specific IgG^+^ RBD^+^ MBCs, could produce antibodies and had an positive cellular response. Interestingly, there were 2 siblings within our cohort with an NFKB1 defect with the presence of positive S-specific IgG antibodies and positive cellular response; in whom, we detected no specific IgG^+^ RBD^+^ MBCs in one of them, similar findings to those reported in the cohort of Amodio et al. in which one of their 2 siblings with the NFKB1 defect lacked humoral response, and both of them presented impaired cellular response (33, 34).

Our study showed that the reduction in B-cell counts in peripheral blood (<100 CD19 B cells/µL) and a reduced class-switched memory B-cell compartment (<2% Sm B cells) are critical for a good humoral response in terms of S-specific IgG antibodies, as others have already reported (30, 34, 35). The presence of B cells in peripheral blood is sufficient to generate specific antibodies in most patients. In certain cases, however, it is insufficient for a proper detection of specific IgG^+^ RBD^+^ MBCs, given that we were able to detect them in only 55% of those patients. Other studies have also reported heterogenic results in their CVID cohort when analyzing specific IgG^+^ RBD^+^ MBCs (33). Interestingly, we found 2 CTLA-4 patients from our cohort without detectable B cells in peripheral blood. These patients were able to produce antibodies after natural SARS-CoV-2 infection or after vaccination. One possible explanation is that these patients had a tendency to a rapid response in generating antibodies by plasma cells in secondary lymphoid organs or bone marrow, without detectable B cells in peripheral blood (9, 11). Another possible explanation found by Salinas et al. who analyzed the B-cell phenotype and found atypical specific B cells in patients with CVID and suggested that it could be the consequence of impaired GC reactions (32).

However, other patients of our cohort without detectable specific IgG^+^ RBD^+^ MBCs, including 1 with X-linked agammaglobulinemia deficiency, 2 with CVID, and a patient with profound hypogammaglobulinemia after rituximab treatment, also presented impaired antibody production but positive T-cell response by using a ELISpot estimating IL-2 and IFNγ secretion (27, 33), as previously reported. This situation is slightly different from those patients that are treated continuously with B-cell-depleting therapies; despite, the separation between treatment and vaccine of almost 20 weeks, only 36% of the patients could generate specific antibodies following vaccination (36).

Within our cohort, we also studied other IEI classified as CID associated with syndromic features, such as Wiskott-Aldrich syndrome, in which a proper humoral and cellular response was detected, as similar results reported (31). In 2 patients with DiGeorge syndrome, we observed a complete humoral response in one and low titers of specific antibodies but with no cellular response in the other, similar results as those obtained by Squire et al. (31). Despite the absence of B cells in peripheral blood, the 2 patients with CTLA-4 in our cohort produced antibodies after natural SARS-CoV-2 infection and vaccination, respectively, and presented positive T-cell response, although other studies have observed a lack of T-cell response in patients with CTLA-4 by using other technique detecting antigen specific T cells (34). In APECED (another IEI disease caused by immune dysregulation), Delmonte et al. found that the percentage of positive humoral response could be reduced in those patients (30). Of note, the APECED patient of our cohort could also generate S-specific IgG antibodies, but we were unable to detect IgG^+^ RBD^+^ MBCs.

We observed a profound failure of B and T-cell response in 3 of the patients studied: one patient with CD40L deficiency classified as CID affecting humoral and cellular immunity; one patient with CGD classified as a congenital defect of phagocyte and one patient with APDS1, a predominant antibody deficiency. Other studies also found undetectable levels of specific antibodies in 3/21 patients with IEI and impaired T-cell response in 5/21 patients (33).

Several studies in general population have raised the importance of booster for a greater protection against SARS-CoV-2 infection and leads to reduce disease severity. Among IEI patients the third doses of BNT162b2 reduces the seroconversion failure from 45% to 26% (37), similar results from Shields et al, reducing the seroconversion failure from 39 to 34% in IEI patients (38). However, there are still some IEI patients that failed to produce antibody response or a T cell response.

IEI patients are a good example to understand molecular mechanism of the immune system. In general, several reports have shown that the risk factors, the severity of the disease and case fatality in patients with IEI was similar to that observed in the general population (39). Also, a review of Tangye et al. (40) nicely summarizes that the percentage of infected asymptomatic IEI patients is similar to general population (10-20%), the majority of IEI patients developed mild disease (30-50%). Although there are some differences with the general population, for example IEI patients have increased case fatality rate, increased admission rate to ICU, younger age of infection and younger age at death, and longer disease duration with longer viremia/viral shedding. Also, similar findings showed in a Brazilian cohort, with a direct relationship between the severity of SARS-CoV-2 infection in IEI patients and older age and higher number of comorbidities (41) and reported by the Italian Primary Immunodeficiencies Network (42). Cousins et al. reported that patients through different phases of the pandemic experiences mild clinical course with limited symptoms (43). And that the clinical impact of COVID-19 in IEI patients varies from mild symptoms to death. However, Cousins et al, also reported that the Mount Sinai cohort over time have been less ill and required less hospitalization, in accordance to general population. Another study in IEI pediatric to young adults, reported higher COVID-19 pneumonia rate that the general age-range population with lower humoral and cellular responses in the acute phase (44).

The burden of the SARS-CoV-2 pandemic for the whole population, with its many initial uncertainties, was even greater for immunocompromised patients. Clinical laboratories in reference centers for IEI immediately assumed the task of exploring and validating novel methodologies to asses antibody and cellular specific responses to natural disease and first vaccines. This was crucial to support, as much as possible, individual data-based clinical decisions and risk assessment for patients. In this article we describe an in-depth evaluation of vaccine responses based on solid laboratory methods. Our results and others suggest that IEI and more specifically PADs patients elicit T cell responses, and remarkably antibody responses to rather similar to healthy controls. These methods could be applied in prospective studies for further delineating the persistence and reinforcement of these responses upon subsequent viral exposures or vaccine boosts.

## Data availability statement

The original contributions presented in the study are included in the article/**Supplementary Material**. Further inquiries can be directed to the corresponding authors.

## Ethics statement

The studies involving human participants were reviewed and approved by Hospital Universitario La Paz, Madrid, Spain (PI-4101). The patients/participants provided their written informed consent to participate in this study.

## Author contributions

ELG conceived the project. ELG, LPM, YBG and PN designed the experiments. LPM, YBG, PN and YS-S performed the experiments. LPM, YBG, PN and ELG wrote the manuscript. All authors contributed to the article and approved the submitted version.
