# Supplementary material for: Detection of specific RBD+ IgG+ memory B cells by flow cytometry in healthcare workers and patients with inborn errors of immunity after BNT162b2 m RNA COVID-19 vaccination
Source: Front Immunol. 2023 May 4;14:1136308. doi: 10.3389/fimmu.2023.1136308 (PMC10192857; doi:10.3389/fimmu.2023.1136308)

**Supplementary**

**S1. Figure 1:** A: Gating strategy used for the classification of circulating B-cell populations. Total B cells were gated first after exclusion of debris and cell doublets (FSC-A/FSC-H), then by their low-to-intermediate forward (FSC) and sideward (SSC) light scatter properties and for the positive staining for CD19. B cells (CD19+) were then separated into subpopulations based on their staining profile for CD38, CD27, surface membrane IgM, IgD, IgG, and IgA. IgMD+ MBCs (memory B-cells), IgG+ MBCs, and IgA+ MBCs. RBD+ expression was analyzed in IgG+ MBCs. Representative dot plots showing the expression of specific RBD+ IgG+ MBCs prior to vaccination and 21 days after the second dose.

**
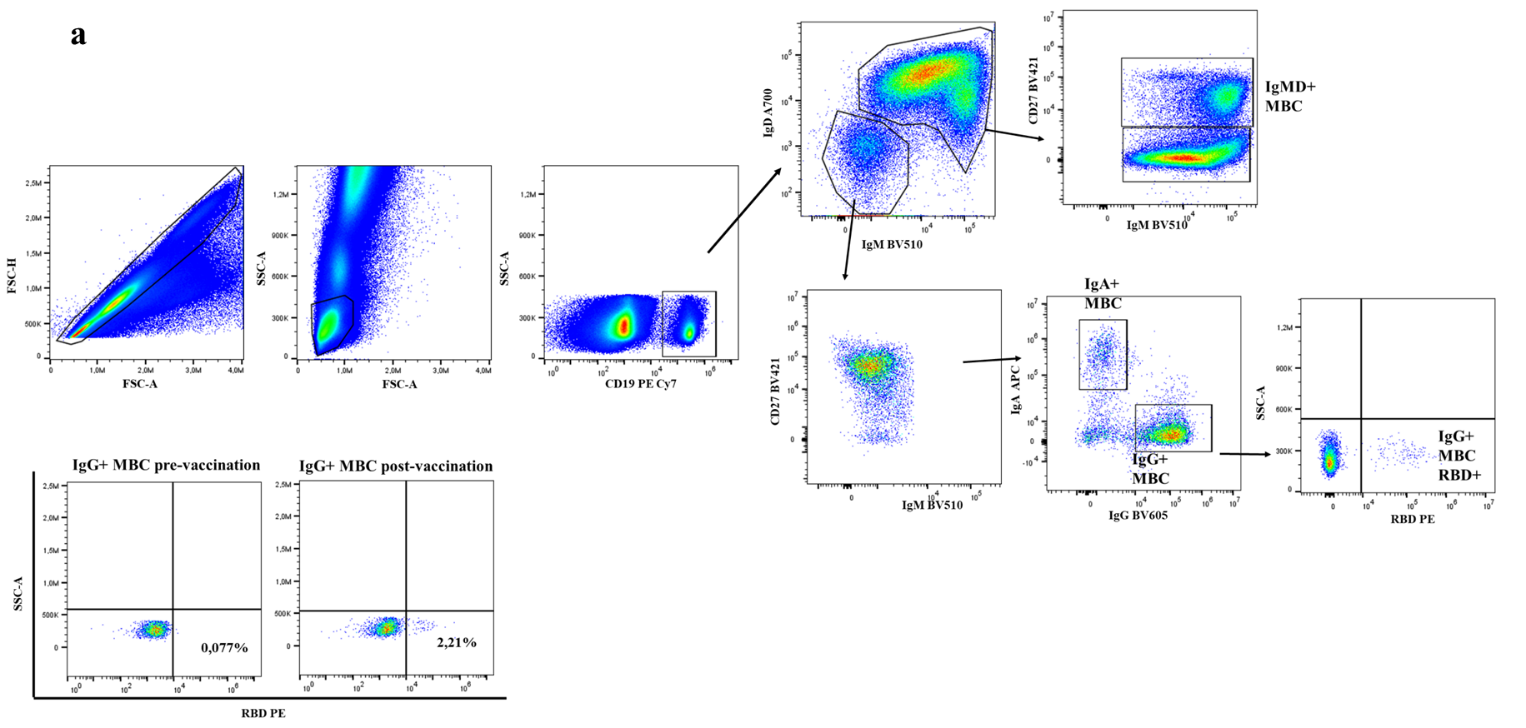
**

**S2. Figure 2:** The percentage of specific RBD^+^ IgG^+^ MBC response to the second dose in those individuals with pre-existing immunity to previous CCC infection exceeds the titers found pre-vaccination.


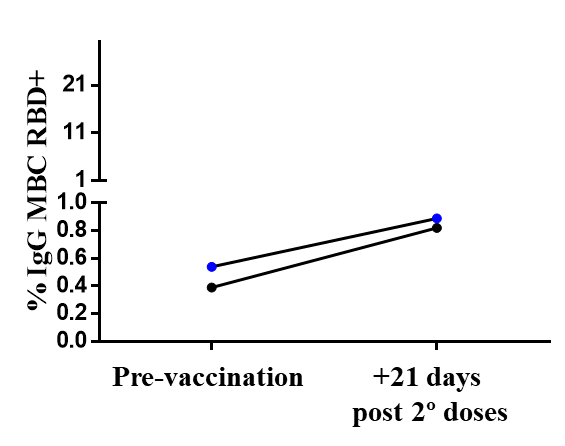

Supplement: Supplementary file 1 [file DataSheet_1.docx]
