# Supplementary material for: Detection of specific RBD+ IgG+ memory B cells by flow cytometry in healthcare workers and patients with inborn errors of immunity after BNT162b2 m RNA COVID-19 vaccination
Source: Front Immunol. 2023 May 4;14:1136308. doi: 10.3389/fimmu.2023.1136308 (PMC10192857; doi:10.3389/fimmu.2023.1136308)
Supplement: Supplementary file 2 [file DataSheet_2.doc]

**Supplementary**

**S1. Table:** List of antibodies for the analysis of B-cells and B-cell subsets by flow cytometry.

| **Analysis specific RBD^+^ B cells** | | | | | | | | |
| --- | --- | --- | --- | --- | --- | --- | --- | --- |
| **Fluorochrome** | **BV421** | **BV510** | **BV605** | **PE** | **Pe-Cy7** | **APC** | **A700** | **APC-H7** |
| **Antibody** | CD27 | IgM | IgG | RBD | CD19* | IgA | IgD | CD38 |
| **Clone** | M-T271 | MHM-88 | G18-145 |  | J3-119 | IS11-8E10 | IA6-2 | HB7 |
| **Manufacturer** | BD Biosciences | Biolegend | BD Biosciences | Immunostep | Beckman Coulter | Miltenyi | Biolegend | BD Biosciences |

**⁕**For patients with CD19 defect, CD20 Pe Cy 7 was used from Exbio (clone 2H7).

**S2. Table:** IEI cohort characteristics including demographics, diagnostic and clinical data. Routine immunological evaluation includes: IgG, IgA, IgM levels, lymphocytes absolute counts, T, B and NK percentages and absolute counts. Absolute counts of naive and memory B and T cell compartments.

F: female; M: male; IgRT: immunoglobulin replacement therapy; Usm: unswitched memory B cells; Sm: switched memory B cells.

(continue)
